# Frequency doubling technology, optical coherence technology and pattern electroretinogram in ocular hypertension

**DOI:** 10.1186/1471-2415-12-33

**Published:** 2012-08-01

**Authors:** Mauro Cellini, Pier Giorgio Toschi, Ernesto Strobbe, Nicole Balducci, Emilio C Campos

**Affiliations:** 1 Department of Specialistic Surgery and Anesthesiology Science, Ophthalmology Service, University of Bologna, Via Palagi 9, Bologna, Italy

**Keywords:** Frequency-doubling technology, Ocular hypertension, Optical coherence tomography, Pattern electroretinogram, Retinal ganglion cells, Retinal nerve fiber layer

## Abstract

**Background:**

To assess which of three methods, namely, optical coherence tomography (OCT), pattern electroretinogram (PERG) or frequency-doubling technology (FDT), is the most sensitive and specific for detecting early glaucomatous damage in ocular hypertension (OH).

**Methods:**

Fifty-two patients with OH (24 men and 28 women, mean age of 56 ± 9.6 years) with an intraocular pressure (IOP) > 21 mmHg and fifty-two control patients (25 men and 27 women, mean age of 54.8 ± 10.4 years) with IOP < 21 mmHg, were assessed. All the patients had normal visual acuity, normal optic disk and normal perimetric indices.

All subjects underwent OCT, FDT and PERG. Data were analyzed with unpaired *t*-tests, Chi-square test and Receiver Operating Characteristic (ROC) curve analyses.

**Results:**

In patients with OH, OCT showed retinal nerve fiber layer (RNFL) thinner than in control group in the superior quadrant (130.16 ± 10.02 vs 135.18 ± 9.27 μm, respectively; p < 0.011) and inferior quadrant (120.14 ± 11.0 vs 132.68 ± 8.03 μm; p < 0.001). FDT showed a significantly higher pattern standard deviation (PSD) (3.46 ± 1.48 vs 1.89 ± 0.7 dB; p < 0.001). With respect to PERG, only the amplitude showed significant differences (p < 0.044) between the two groups. ROC curve analysis revealed a sensitivity and specificity of 92% and 86%, respectively, for FDT-PSD (with an area under the ROC curve of 0.940), whereas with OCT, a sensitivity of 82% and a specificity of 74% was recorded in the inferior RNFL quadrant (with an area under the ROC curve of 0.806) finally with PERG amplitude we found a sensitivity of 52% and specificity of 77% (with an area under the ROC curve of 0.595).

**Conclusions:**

FDT is the most sensitive and specific method for detecting early glaucomatous damage in eyes with OH, and together with OCT, can be useful in identifying those patients who may develop glaucoma.

**Trial registration:**

ISRCT number: ISRCTN70295497

## Background

Glaucoma is an optic neuropathy characterized by the progressive loss of ganglion cells and visual field alterations. Systematic review of all population based surveys on blindness and low vision in 2006 estimated 60.5 million people with glaucoma worldwide in 2010, with bilateral blindness in 8.4 million people [1]. The appearance of alterations in the retinal nerve fiber layer represents an early sign of glaucoma damage that precedes changes in the optic nerve and peripheral alterations [2].

The current quantitative techniques used for examination of the retinal nerve fiber layer (RNFL) are scanning laser polarimetry (SLP) [3], Heidelberg retinal tomography (HRT) [4] and, more recently, optical coherence tomography (OCT) [5]. OCT makes it possible to examine high-resolution cross-sections of ocular tissues using the principle of low coherence interferometry [6,7] and seems more reliable than SLP and HRT for the evaluation of the RNFL [8].

Due to the irreversible nature of the retinal ganglion cell loss and axonal damage in glaucoma, it is particularly important to use high-quality investigative techniques to facilitate the early detection of functional deterioration in susceptible patients [9]. The qualitative analysis methods currently available are standard achromatic perimetry (SAP) and short wavelength automated perimetry (SWAP) [10,11], as well as the recent frequency-doubling technology (FDT) perimetry [12]. The pattern electroretinogram (PERG) was introduced 25 years ago as a method to discriminate between healthy and glaucomatous eyes by recording the electrical potentials of retinal ganglion cells [13-15]. Ganglion cell damage is the main cause of decreased visual sensitivity in glaucomatous eyes [16,17]. In this context, this study assessed which of the three methods, OCT, FDT or PERG, is the most sensitive and specificity for the early detection of glaucomatous damage in a group of patients with ocular hypertension (OH), the most widely recognized risk factor for progression of glaucomatous damage.

## Methods

The OCT, FDT and PERG examinations of 52 patients, consisted of 28 women and 24 men aged between 44 and 76 years (mean 56 ± 9.6 years), were analyzed. All patients had an intraocular pressure (IOP) greater than 21 mmHg (mean 23.96 ± 1.3 mmHg) and with no other ocular (e.g., cataracts or other opacities) or systemic diseases. The patients all had normal visual acuity (VA), a normal optic disk (in particular, with no sign of diffuse thinning or focal narrowing or notching of the neuroretinal rim, hemorrhage, cupping or visible or progressive changes in the fiber layer on ophthalmoscopic examination with a +78 diopter lens) and mean defect (MD) and pattern standard deviation (PSD) perimetric indices of less than 1.5 dB (0.28 ± 1.1 and 0.65 ± 0.4, respectively). All patients were recruited from the Glaucoma Service of the S. Orsola-Malpighi Hospital of Bologna.

The control group consisted of 55 subjects: 28 males and 27 females aged between 42 and 75 years (mean 54.8 ± 10.4 years). The healthy controls had no ongoing eye or systemic disorders nor such a history, an IOP of less than 21 mmHg, a normal optic nerve and normal visual field indices.

A normal visual field was defined by the absence of each of these responses: a cluster of 3 points lower than P < 5% or a cluster of 2 points lower than P < 1% on a pattern deviation plot, or PSD with P < 5%.

Only one eye per subject, both in the OH and the control groups, was randomly chosen if both eyes were eligible for the study.

The study was approved by the Local Ethics Committee of the S. Orsola-Malpighi Hospital, Bologna. An oral informed consent was obtained from all the patients.

All patients underwent an ophthalmologic examination including visual acuity, applanation tonometry IOP assessment, corneal radius curvature measurement with automated keratometry (RK, Canon Inc., Tokyo, Japan), corneal thickness evaluation with a Tomey SP3000 pachymeter (Tomey Corp., Nagoya, Japan), biomicroscopy of the anterior and posterior segment with automatic measurement of the cup/disc (C/D) area ratio of the optic nerve head with an Stratus OCT 3 (Zeiss-Humphrey, Dublin, CA, USA). SAP was also performed with a Humphrey Field Analyzer-30.2, using the full-threshold program (Zeiss-Humphrey, Dublin, CA, USA). Three visual field tests were performed for each OH and healthy control individual, and only the results of the third test were assessed. All subjects underwent assessment of the RFNL with the Stratus OCT 3 (Zeiss-Humphrey, Dublin, CA), FDT perimetry with the FDT Visual Field Instrument (Welch-Allin FDT, Skaneatsles Falls, NY, USA and CarlZeiss, Meditec Inc., Dublin, CA, USA), and PERG with the RetimaxPlus system (CSO Instruments, Florence, Italy). For FDT, only the results of the third test were assessed, and the FDT and SAP were reviewed separately by two investigators who were blinded to any clinical data.

### OCT scanning

The OCT technique makes it possible to analyze the retinal structures and obtain in vivo tomographic sections illustrating the histological retinal structure. This instrument use low coherence interferometry principles, which separate the retinal microstructures by measuring the echo delay of the light reflected and retrodiffused by these structures. The OCT 3 makes it possible to obtain scans with an axial resolution of 10 μm and a transverse resolution of 20 μm. The instrument projects onto the retina a beam of light generated by a superluminescent diode, with a infrared wavelength (820 nm). The system detects, processes and stores the retinal delay patterns and displays and stores the selected scans so that they can be subsequently analyzed. Each eye was dilated with 1% tropicamide before recording the images, and internal fixation was chosen because it provides better reproducibility than external fixation.

This instrument was used to directly measure the thickness of the peripapillary RNFL using the RNFL Thickness Averaging program, in which three consecutive, circular scans are performed, each one 3.4 mm in diameter and centered on the optic nerve. The thickness of the fibers was defined as the number of pixels obtained between the anterior and posterior RNFL projection. The values obtained with each scan are displayed in graphical form, resembling a clock face divided into four quadrants, representing the superior, nasal, inferior and temporal sections of the RNFL expressed in microns.

### FDT perimetry

FDT perimetry is a new technique designed for the rapid and effective identification of visual field impairment due to glaucoma. The FDT stimulus consists of a bar grid with a low-frequency spatial sinusoidal profile (0.25 cycles/degree) subjected to a sinusoidal temporal commutation at a frequency of 25 Hz. FDT is based on the principle of the frequency-doubling illusion, in which the subject perceives twice the number of bars actually presented [18]. The cells that present a nonlinear response to the contrast in the test image, which are therefore responsible for this illusion, are a subgroup of M cells [19].

FDT was performed using the full-threshold program N-30. With this test, target stimuli consisted of individual sinusoidal gratings, 10 degrees square at 0.25 cycles/degree, alternately flashing at 25 MHz. Targets were in one of the 19 areas within the central 30 degrees of the visual field. For each visual field, we evaluated the mean defect (MD) and the pattern standard deviation (PSD).

### PERG recording

For PERG recording, we followed the International Society for Clinical Electrophysiology of Vision (ISCEV) standard guidelines [20].

The patient sat in a chair at a distance of 114 cm from the television screen. The generated potential was measured with skin electrodes. The ground electrode was placed on the right ear lobe, and the interelectrode resistance was less than 3 kW. The PERG stimulus was a black-and-white checkerboard with a contrast of 99% at 1.6 cycles/degree, four reversal/s and a mean luminance of 110 cd/m^2^. The monitor screen subtended a visual angle of 12.5°. The refraction of all subjects was corrected for the viewing distance. No mydriatic or miotic drugs were used. The transient PERG response was characterized by three subsequent peaks that, in normal subjects, are indicated on the basis of polarity and latency: N35, P50 and N95. The P50 amplitude was measured from the trough of N35 to the peak of P50. In some patients, the N35 was poorly defined; in these cases, N35 was replaced with the average between time zero and the onset of P50.

### Statistical analysis

All statistical analyses were performed using the FASTAT Version 2 software package (Systat Inc., Evanston, Illinois). Unpaired student’s *t*-test was used taking p < 0.05 as significant. The Chi-square test was used for categorical data. The Receiver Operating Characteristic (ROC) curve analysis was performed to determine the diagnostic sensitivities and specificities of OCT, FDT, and PERG. To compare ROC curve areas, Delong test was used.

## Results

The demographic and ocular characteristics of the OH patients and healthy controls are presented in Table 1.

**Table 1:** Demographics data for OH patients and controls

	**Controls**	**Ocular Hypertension**	**p < 0.05**
Male: Female	27:25	24:28	0.765*
Age (years)	54.8 ± 10.4	56 ± 9.6	0.431
IOP (mmHg)	16.7 ± 1.5	23.96 ± 1.3	0.001
Refractive errors (diopters)	- 0.2 ± 1.5	- 0.4 ± 1.6	0.320
Visual acuity (LogMAR)	0.0 ± 0.1	0.0 ± 0.1	1.00
Corneal radius (mm)	7.76 ± 0.14	7.83 ± 0.14	0.210
Corneal thickness (μm)	552.3 ± 3.08	558.4 ± 4.15	0.274
C/D area ratio	0.33 ± 0.11	0.37 ± 0.12	0.068
Visual field MD (dB)	0.28 ± 1.1	0.31 ± 1.2	0.670
Visual field CPSD (dB)	0.65 ± 0.4	0.90 ± 0.5	0.542

Demographics data of intraocular pressure (IOP), cup/disc area ratio (C/D), visual field mean defect (MD), visual field corrected pattern standard deviation (CPSD), “*” Chi-square test for ocular hypertensive (OH) patients and controls.

No significant differences in clinical and epidemiologic factors were found between the two groups, except for IOP (p < 0.001). The other predictive factors for the development of glaucoma (age, cup-disc ratio and central corneal thickness) were similar in the two groups.

**Figure 1:**
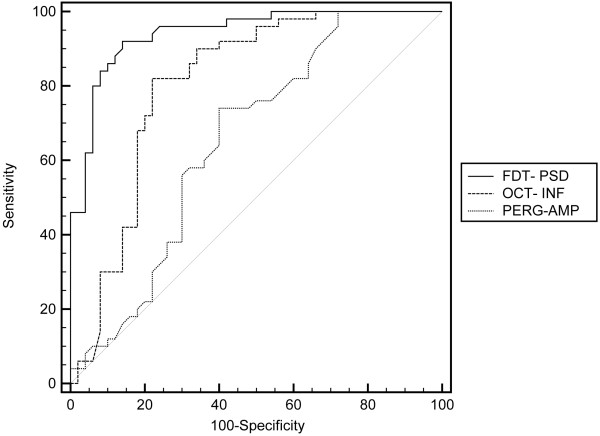
The ROC curves of the indices, doubling frequency technology (FDT) pattern standard deviation (PSD), pattern electroretinogram (PERG) amplitude and optical coherence tomography (OCT) evaluation of the inferior retinal nerve fiber layer (RNFL), with the highest sensitivity/specificity ratio.

OCT revealed RNFL thinning in all quadrants in the OH patients compared with the healthy controls, but this was significant (p < 0.05) only for the superior and inferior quadrants. FDT showed only a significantly higher PSD (p < 0.001) in OH patients than in healthy controls (Table 2). Regarding PERG, we found a decreased amplitude (p < 0.044) in the OH patients, whereas for latency, no significant differences were found between the two groups. ROC curve analysis revealed a sensitivity and specificity of 92% and 86%, respectively, for FDT-PSD, whereas for OCT, a sensitivity of 82% and a specificity of only 74% was recorded in the inferior RNFL quadrant. Finally the PERG amplitude had a sensitivity 52% of and specificity of 77% (Table 3 and Figure 1). OCT measured inferior quadrant RNFL thickness and FDT PSD discriminated between OH and healthy eyes better than PERG amplitude (p = 0.04 and <0.0001, respectively). Moreover, FDT PSD discriminated between OH and healthy eyes better than OCT measured inferior quadrant RNFL thickness (p = 0.01).

**Table 2:** Mean values for RNFL thickness, FDT perimetric indices and PERG P50 amplitudes and latency values in OH patients and controls

	**Controls**	**Ocular Hypertension**	**p < 0.05**
RNFL superior (μm)	135.18 ± 9.27	130.16 ± 10.02	0.011
RNFL inferior (μm)	132.68 ± 8.03	120.14 ± 11.01	0.001
RNFL nasal (μm)	82.97 ± 7.15	81.30 ± 3.74	0.146
RNFL temporal (μm)	85.97 ± 5.92	84.42 ± 3.66	0.118
FDT-MD (dB)	−1.78 ± 0.75	−2.09 ± 1.29	0.145
FDT-PSD (dB)	1.89 ± 0.70	3.46 ± 1.48	0.001
PERG amplitude (μVolt)	1.70 ± 0.70	1.52 ± 0.25	0.044
PERG latency (ms)	55.13 ± 2.24	55.85 ± 4.12	0.139

Mean values for retinal nerve fiber layer (RNFL) thickness obtained with optical coherence tomography (OCT), frequency doubling technology (FDT) mean defect (MD) and pattern standard deviation (PSD) perimetric indices and pattern electroretinogram (PERG) of the wave P50 amplitudes and latency values in OH patients and controls.

**Table 3:** The area under ROC curve

	**Sensitivity**	**Specificity**	**AUC (95% CI)**
RNFL superior	56%	76%	0.510 (0.391 to 0.630)
RNFL inferior	82%	74%	0.806 (0.717 to 0.898)
RNFL temporal	58%	70%	0.633 (0.518 to 0.748)
RNFL nasal	60%	52%	0.575 (0.458 to 0.691)
MD-FDT	48%	74%	0.528 (0.412 to 0.644)
PSD-FDT	92%	86%	0.940 (0.876 to 0.979)
PERG amplitude	52%	77%	0.654 (0.552 to 0.746)
PERG latency	42%	72%	0.593 (0.480 to 0.707)

Percentage sensitivities and specificities of retinal nerve fiber layer (RNFL) thickness, frequency doubling technology (FDT) mean defect (MD) and pattern standard deviation (PSD) and pattern electroretinogram (PERG) amplitude and latency area under Receiver Operating Characteristic (ROC) curve. AUC = area under the curve; CI = confidence interval.

## Discussion

Glaucoma is the second cause of blindness worldwide [1] and an early diagnosis is important to treat it promptly in order to avoid or reduce the progression of visual field defects. At least 25 to 30% of RGC is already lost when visual field defects are detectable by SAP [21]. Elevated IOP is the most important risk factor for glaucoma onset and progression [22], but the treatment of only high-risk patients with OH should be considered [23]*.* So, it is important to identify those patients with elevated IOP who have early stage glaucoma in order to decide when patient need a therapy.

In our study OCT of the retinal nerve fiber layer revealed a significant reduction in thickness in the inferior quadrant of the optic disc in OH compared with healthy controls, with a sensitivity of 82% and a specificity of 74%. Several studies suggested that optic nerve and RFNL impairment can generally be identified before SAP alterations [24-26]*.* We could speculate that at the onset of glaucoma, only the peripheral visual field is impaired, and thus, the initial sight deficit is not detected by the automated techniques currently in use [27]. OCT morphology correspond well to histopathological findings [28]. The RNFL thinning observed in our OH patients confirms previous studies, where different methods of analysis were used, like OCT [29], scanning laser polarimetry [30] or confocal scanning laser ophthalmoscope [31,32].

Furthermore FDT shows a significant increase in the PSD index in hypertensive eyes, with a sensitivity of 92% and a specificity of 86%. FDT is a highly sophisticated method, that examine the functionality of a subgroup of magnocellular ganglion cells [12], called My cells, that represent just 3% of all retinal ganglion cells [33,34]. Histopathological experimental studies of optic nerve in glaucoma patients suggest an early and selective impairment of M-cells [35,36]. The percentage of our OH eyes with abnormal FDT results is comparable with other studies [26,37-40], but this elevated percentage could be partially due to false positive results.

Finally with transient PERG, a reduction in P50 amplitude was found in 78% of OH patients, with a sensitivity of 52% and a specificity of 77%, whereas an increase in latency was found in only 62% of cases. These data are similar to findings of previous studies, were steady-state PERG was employed [27,41,42]. PERG measures RGC functional activity and is correlated with the number of functioning cells [43,44]. PERG alteration in OH subjects would confirm that in OH the damage is localized to the RGC [45,46] and that RGC dysfunction precedes their death [44]. PERG amplitude is inversely related to IOP in OH group [47]. So, we could suppose that PERG amplitude difference between OH and control group could be in part due to different IOP values and not to early disease. Furthermore, the low sensitivity of PERG, which does not exceed 0.52, in detecting functional damage to RGC in OH when compared with OCT and FDT, may be related to the fact that the test stimulus is central in PERG, whereas glaucomatous impairment initially affects the peripheral visual field [27]. Instead, FDT might be more sensitive to peripheral defects because of the distribution of magnocellular cells. Furthermore, PERG reflects diffuse, non-focal damage to ganglion cells [48], so the initial focal damage could not be detected and ocular opacities may also decrease PERG amplitude [49].

This study has several limitations: first, it is retrospective and this could influence the results. Second, this is a glaucoma detection study, but the lack of a gold standard for glaucoma detection makes it difficult to compare different tests. Third, we evaluated 52 OH patients and 55 controls, but a larger sample size could improve the diagnostic accuracy of the study.

## Conclusions

Our study demonstrates that FDT is slightly more sensitive and more specific than OCT in highlighting nerve fiber alterations in OH. The relatively low sensitivity of OCT may reflect the fact that this technique, which uses coherent light, can be influenced by the opacity of the cornea, lens and the vitreous humor. PERG is also a useful diagnostic technique, although it entails the limitations inherent to any experimental method, as the procedures used vary considerably between one laboratory and another, making it more difficult to standardize and reproduce than OCT and FDT.

Thus, from a clinical point of view, we think that the current examination of RNFL thickness using OCT and the study of RGC functionality with FDT could be very useful for identifying patients at risk for developing glaucomatous optic neuropathy.

## Competing interests

The authors declare that they have no competing interests.

## Authors’ contributions

MC drafted the manuscript, PGT recruited the patient from the Glaucoma Service of the S.Orsola-Malpighi Hospital, ES made OCT and FDT exams, NB made PERG exam and EC review the manuscript. All authors read and approved the final manuscript.

## Pre-publication history

The pre-publication history for this paper can be accessed here:

http://www.biomedcentral.com/1471-2415/12/33/prepub
